# Seroprevalence and factors associated with bovine and swine toxoplasmosis in Bobo-Dioulasso, Burkina Faso

**DOI:** 10.14202/vetworld.2019.1519-1523

**Published:** 2019-10-02

**Authors:** Dieudonné Tialla, Laibané Dieudonné Dahourou, Oubri Bassa Gbati

**Affiliations:** 1 Département Biomédical et Santé Publique, Unité des Maladies à Potentiel Epidémique, Maladies Emergentes et Zoonoses, Institut de Recherche en Sciences de la Santé, Bobo-Dioulasso, Burkina Faso; 2 Department of Animal Health and Veterinary Public Health, Ecole Nationale d’Elevage et de Santé Animale, Ouagadougou, Burkina Faso; 3 Department of Sciences and Technologies, Université Saint Thomas d’Aquin, Ouagadougou, Burkina Faso; 4 Department of Livestock and Animal Production, Environmental Sciences and Rural Development Institute, University of Dedougou, P.O. Box 174, Dedougou, Burkina Faso; 5 Department of Public Health and Environment, Interstate School of Veterinary Science and Medicine, P.O Box 5077, Dakar, Senegal

**Keywords:** Burkina Faso, cattle, public health, seroepidemiologic studies, swine, toxoplasmosis

## Abstract

**Background and Aim:**

Toxoplasmosis is a worldwide zoonosis with major public health importance. To know more about this condition in Burkina Faso, this study was implemented to determine the seroprevalence of *Toxoplasma gondii* infection among pigs and cattle in intra-urban and peri-urban area of Bobo-Dioulasso (Burkina Faso).

**Materials and Methods:**

Serum samples were collected from 600 cattle and 600 pigs with 300 samples from each species in intra-urban and peri-urban area of Bobo-Dioulasso. Data about age, sex, and breed of each animal were also noted. Serum samples were analyzed by indirect enzyme-linked immunosorbent assay to look for immunoglobulin G and immunoglobulin M antibodies to *T. gondii*.

**Results:**

This study revealed a herd prevalence of 92.5% and 75%, respectively for porcine and bovine toxoplasmosis. At the individual level, we found a prevalence of 29% and 49.2% for cattle and pigs, respectively. For each species, we noticed a significant association between age, sex, breed husbandry system, and the presence of anti-*T. gondii* antibodies. The prevalence was significantly higher in female, intra-urban system, exotic breed, and animal <2 years old (p<0.05)

**Conclusions:**

The results provided evidence for the presence of *T. gondii* in pigs and cattle farms around Bobo-Dioulasso. Hence, in Bobo-Dioulasso, raw or undercooked meat consumption is a risk for *T. gondii* infection for human. Knowledge of the prevalence of toxoplasmosis will help to target prevention efforts.

## Introduction

Toxoplasmosis is a major zoonosis caused by the intracellular protozoan named *Toxoplasma gondii*, which affects humans and many animals. It is of medical, economic and social importance, and public health concern. *Felidae*, especially the cat, is the only definitive hosts known [1] and they release millions of parasite oocysts in the environment. These oocysts after sporulation can maintain their infectivity for several months in water and soil and are responsible for the infestation of intermediate hosts including man and other animals [2]. Man and these animals acquired toxoplasmosis when they ingested raw or undercooked meat of infected animals [3-5] or food or water contaminated by oocysts [3,6]. It is usually asymptomatic in immunocompetent individuals but can lead to serious complications in immunosuppressed individuals. There is also congenital transmission which leads to serious neurological or ocular disorders with cardiac and brain abnormalities such as hydrocephalus, mental retardation, seizures or fetal mortality [7,8]. It is common in pregnant women and can lead to abortions [9].

In Burkina Faso, studies on toxoplasmosis were done on sheep [10], pig carcasses [11], and chickens [12]. However, data on the epidemiology of toxoplasmosis in live pigs and cattle are non-existent, while the meat of these two species is highly consumed in the country and could constitute, especially for pork [13,14] an important source of transmission of toxoplasmosis to humans.

Thus, this study aimed to evaluate the prevalence of bovine and porcine toxoplasmosis in intra-urban and peri-urban farms of Bobo-Dioulasso city.

## Materials and Methods

### Ethical approval and informed consents

First, to each sampling, the aim of this study was explained to farmers. Farmers who accepted to participate in this study signed an inform consent letter before each animal sampling. This study also received approval clearance (No.2016-15/MS/SG/CM/CEI) from Centre Muraz Ethical Committee. Moreover, all blood samples were collected by well-trained veterinarians with respect to animal welfare.

### Study area

This study was implemented from August 2016 to July 2017 in the intra- and peri-urban area of Bobo-Dioulasso, the capital city of Hauts-Bassins region. Bobo-Dioulasso is the second largest city of Burkina Faso and is located in the south of the country. It is located at 11° 10′ 38″ North and 4° 17′ 52″ West. The laboratory assays were done in the unit of potential epidemic diseases, emerging diseases, and zoonosis of Institut de Recherche en Sciences de la Santé at Bobo-Dioulasso.

### Study population and sampling method

This study involved pig and cattle populations around Bobo-Dioulasso city. First, we did a preliminary survey to identify the number of cattle and pig farms in and around Bobo-Dioulasso city. For this study, only farms with at least 15 animals were included in the study. During this survey, we identified 62 pig farms and 53 cattle farms. Then, 40 pig farms and 40 cattle farms were randomly selected. In these chosen farms, 600 pigs and 600 cattle were randomly selected. In each farm, two visits were made. The first aimed at sensitizing farmers about our study and the second was focused on animal sampling, blood collection, and questionnaire survey. During animal selection step, 10-20 animals were selected in each farm according to farms according to the size of the herd.

### Data collection

After proper restraint of each selected animal, 4 ml of blood were collected from the jugular vein using a sterile needle. Sterile labeled (Farm and animal code) vacutainer tubes without anticoagulant were used to collect blood from animals. Blood samples were transported to the laboratory with an ice pack and centrifuged at 3000 rpm for 10 min for serum collection. The serum collected was kept in a labeled Eppendorf tube and stored in the freezer at −20°C until laboratory assays were run.

We also made a questionnaire survey with closed-ended questions. This survey was administered in 15 min interviews in French or local language (Dioula or Mooré). Data collected during this survey were the husbandry system in the farm, the age, sex, and breed of each sampled animal.

### Serology

The sera samples were tested using commercial indirect enzyme-linked immunosorbent assay (ELISA) Kits (ID Screen^®^ Toxoplasmosis Indirect Multi-Species from ID Vet Innovative Diagnostic) to determine the presence of toxoplasma-specific immunoglobulin G antibodies, resulting from infection with T *. gondii*. A 96-well plate was prepared to contain the test and control specimens, before transferring them into the ELISA microplate using a multichannel pipette. Optical densities (OD) were read at 450 nm using microplate reader (Thermo SCIENTIFIC Multiskan GO Version 1.00.38). The test was validated if the mean value of the positive control OD (OD positive [ODP]) was >0.350 (ODP control [ODPC] >0.350), and the ratio of the mean OD values of the positive and negative controls (ODP and OD negative [ODN]) was >3.5 (ODP/ODN >3.5). According to the manufacturer’s recommendation, positivity percentage (PP) was calculated using the formula: PP=([OD sample/ODPC]*100). Samples with PP ≤40% were considered negative, between 40 and 50% were considered doubtful, and ≥50% were considered positive. Doubtful samples were repeated for certainty.

### Statistical analysis

Statistical analyses were performed using R software (R Core Team, Vienna, Austria). The prevalence was calculated using the formula P=NP/N, where P is the prevalence, NP is the number of positive animals, and N is the total number of considered animals. Data were presented as the prevalence±confident interval at 95%. The confident intervals were calculated as described by Martin *et al*. [15]. For statistical analysis, the dependent variable was the positivity of the test, and independent variables were age class, husbandry system, animal breed, and sex. For age, age classes were defined as follows: Class 1: 0-2 years; Class 2: 2-5 years, and Class 3: >5 years. For breed, animals were also grouped in two categories with local breed and exotic breed. To identify the association between the positivity to the test and other variables, a Chi-square (χ^2^) test or Fischer Exact was used. The differences were considered statistically significant when p<0.05.

## Results

### Characteristics of sampled animals

For this study, two husbandry systems (peri- and intra-urban) were identified. The peri-urban system was modern with high investment, and the intra-urban system was a free-roaming system. This study was implemented on 20 farms of each system for cattle and pigs. The local cattle breed was mainly zebu Peuhl soudanien, zebu Goudali, and zebu Azawak; the exotic breed was zebu Gyr et metis. With pigs, we identified national, local breed and exotic breeds such as Large White and metis as Khorogo breed. All data about the sampled animals’ characteristics are reported in Table-1.

**Table 1:** Characteristics of sampled animals in intra-urban and peri-urban areas of Bobo-Dioulasso, Burkina Faso, 2017.

Variables	No. tested	Pigs	Cattle	
	
		Frequency (%)	No. tested	Frequency (%)
Husbandry system				
Peri-urban	300	50	300	50
Intra-urban	300	50	300	50
Age class (years)				
0-2	324	54	72	12
2-5	228	38	246	41
>5	48	8	282	47
Sex				
Males	144	24	132	22
Females	456	76	468	78
Breed				
Local	102	17	252	42
Exotic	498	83	348	58

### Serological results in pigs

Results for the prevalence of swine toxoplasmosis are reported in Table-2. The herd prevalence was 92.5%. The prevalence of porcine toxoplasmosis at the individual level was 49.2±4%. The highest prevalence was noted in pigs <2 years old (70.4%), and the lowest was in local breed pigs (13.7%). This prevalence was significantly associated with sex, age class, breed, and husbandry system (p<0.05).

**Table 2:** Prevalence of *Toxoplasma gondii* antibodies in pigs in intra-urban and peri-urban areas of Bobo-Dioulasso, Burkina Faso, 2017.

Variables	No. tested	No. positive	Prevalence *(%) and 95% CI	p-value
Sex				0.01
Male	144	27	18.7±6.3^a^	
Female	456	268	58.8±4.5^b^	
Total	600	295	49.2±4	
Husbandry system				0.02
Peri-urban	300	94	31.3±5.2^a^	
Intra-urban	300	201	67±5.3^b^	
Total	600	295	49.2±4	
Age				0.01
0-2	324	228	70.4±4.9^a^	
2-5	228	59	25.9±5.6^b^	
>5	48	8	16.7±10.5^c^	
Total	600	295	49.2±4	
Breed				0.02
Local	102	14	13.7±6.6^a^	
Exotic	498	281	56.4±4.3^b^	
Total	600	295	49.2±4	

* Within each variable, prevalence rates with different superscripts are statistically different (p<0.05). CI=Confidence interval

### Serological results in cattle

In cattle, the prevalence of toxoplasmosis at the herd and the individual level were, respectively, 75% and 29±3.6%. This prevalence was significantly higher in animals <2 years old, in intra-urban husbandry system, in females, and in exotic breed animals (Table-3). The highest prevalence was found in animal <2 years old (94.4±5.3%) and the lowest was in males (6.1±4.1).

**Table 3:** Prevalence of *Toxoplasma gondii* antibodies in cattle in intra-urban and peri-urban areas of Bobo-Dioulasso, Burkina Faso, 2017.

Variables	No. tested	No. positive	Prevalence *(%) and 95% CI	p-value
Sex				0.01
Male	132	8	6.1±4.1^a^	
Female	468	166	35.5±4.3^b^	
Total	600	174	29±3.6	
Husbandry system				0.01
Peri-urban	300	39	13±3.8^a^	
Intra-urban	300	135	45±5.6^b^	
Total	600	174	29±3.6	
Age				0.02
0-2	72	68	94.4±5.3^a^	
2-5	246	56	22.8±5.2^b^	
>5	282	50	17.7±4.4^c^	
Total	600	174	29±3.6	
Breed				0.01
Local	252	19	7.5±3.2^c^	
Exotic	348	155	44.5±5.2^d^	
Total	600	174	29±3.6	

* Within each variable, prevalence rates with different superscripts are statistically different (p<0.05). CI=Confidence interval

## Discussion

For this study, we used the serological method because of the diagnosis of toxoplasmosis by the demonstration of *T. gondii* in tissue not easy for epidemiological studies. Therefore, the detection of antibody response by a screening of animals’ sera appears to be the conclusive tool for proper surveillance of toxoplasmosis. According to the manufacturer, the test has a specificity of 100% and a sensitivity of 98.36% and does not cross-react with other coccidian parasites.

In pigs, the prevalence of 49.2±4% in higher than the reported prevalence of 29% in Burkina Faso [11], 29.14% in Nigeria [16], 22.8% in Madagascar [17], using ELISA, 32.10% in Ethiopia [18] using direct agglutination test, 5.2% in Japan [19] using latex agglutination test, 9.8% in Portugal [20] with a modified agglutination test; 19.5% in Brazil [21] using indirect immunofluorescence. In cattle, the prevalence of 29% was higher than 8.26% in Algeria [22] with IFAT, 13.20%in Sudan [23], 13.8% in Nigeria [16] using ELISA, 12.6%in Senegal [24] using Modified agglutination test. These differences may be explained by the mode, and husbandry conditions of animals, climate, abundance, and routine deworming of cats and characteristics of tests used [25].

Prevalence was higher in pigs than in cattle. This is in agreement with Tonouhewa *et al*. [25] who reported that the lowest prevalence rate of toxoplasmosis in African animals and noted in cattle. This lower prevalence in cattle could be associated with the greater resistance of cattle to toxoplasmosis [26]. It could also be explained by the lower density of cats around cattle farms according to Albuquerque *et al*. [27], the high density of cats around a farm is associated with a higher prevalence of toxoplasmosis. The prevalence of toxoplasmosis was significantly higher in the intra-urban system than in the peri-urban system (p<0.05). This was also observed in Zimbabwe, where extensive pigs had the highest prevalence [28]. This can be explained by the lack of hygiene observed in the intra-urban farms, and *T. gondii* oocysts survival is prolonged by several months in stagnant water, slurry, and in substrates such as hay, dust, and barriers of pens [4]. In addition, the presence of cats living permanently near the farms was noted in intra-urban system in Bobo-Dioulasso. Indeed, cats being the definitive hosts of toxoplasmosis will through their feces contaminate animals living areas by oocysts of the parasite. Furthermore, we have noted many younger cats during the survey in the intra-urban system, and Demard [29] noted that young cats excrete oocysts in large numbers during their first infection. These cats can defecate anywhere in buildings and especially in feed and animals could become contaminated during ingestion [4]. To this, must be added the free-roaming of animals. In the intra-urban system, animals wander in the city and can easily be in contact with parasite oocysts and thus easily contract *T. gondii* infection.

The prevalence of infection was higher in animals younger than 2 years compared to animals older than 2 years (p<0.05) as found in Sudan [23] and Iran [30]. However, opposite results noticed in Nigeria [16,25]. As described by Dubey and Beattie [31], animals do not keep antibodies, from colostrum or post-infection, all their life and the antibodies disappear as the animal gets older.

Females had a higher prevalence of infection than males (p<0.05) as found in Nigeria [16], while opposite conclusions in Burkina Faso [11]. According to Alexander and Stinson [32], females are most susceptible to protozoan infestation compared to males. Furthermore, in this study, we have noted lower numbers of males in farms. Most of the time, farmers sell males and keep only a few numbers of males for reproduction and the other males are sold. Furthermore, in our study, the exotic breeds of both pigs and cattle were more infected then the local breed, but Onyiche and Ademola [16] found that the local breeds in Nigeria were more infected. The results in our study could be associated with the lower resistance of exotic breed to pathogens and also different husbandry systems in the study areas which affect the exotic breed resistance to infestation. Furthermore, in Bobo-Dioulasso, the exotic breeds were kept in an intensive system where the density of cats was high.

## Conclusion

Our study showed that antibodies of *T. gondii* are present in surveyed farms animals with a prevalence of 49.2% in pigs and 29% in cattle. Thus, animals from these farms could be a risk factor for the transmission of *T. gondii* to humans through their meat and thus a public health concern. According to our findings, strategic control actions must be implemented to protect people from zoonotic disease.

## Authors’ Contribution

DT: Prepared the questionnaire for data collection and sampling method, made statistical analysis; LDD: Collected sample and made laboratory assay. DT, LDD and OBG: Prepared and revised the manuscript. All authors read and approved the final manuscript.
